# Restorative justice in a case of traumatic birth following an unperceived pregnancy

**DOI:** 10.1007/s00737-023-01416-y

**Published:** 2023-12-28

**Authors:** Ibone Olza, Vanessa Cascante Alfaro, Claudia M. Klier

**Affiliations:** 1 European Institute of Perinatal Mental Health, Madrid, Spain; 2 Edificio Central, Avenida Segunda, 225m, Este de La C.C.S.S, San José, Costa Rica; 3 Department of Child and Adolescent Medicine, CCP Comprehensive Center of Pediatrics, Medical University of Vienna, Währinger Gürtel 18-20, 1090 Vienna, Austria

**Keywords:** Denial of pregnancy, Unperceived pregnancy, Restorative justice, Obstetric violence, Dissociative disorder, Neonaticide

## Abstract

**Purpose:**

Pregnancy can be denied or better “unperceived” by women in up to 1:300 pregnancies and poses the mother and her unborn at high risk when an unassisted birth follows. The importance of recognizing unperceived pregnancy and the risk of unassisted births for both mothers and their babies are described.

**Methods:**

Description of a case of unperceived pregnancy and traumatic unassisted birth.

**Results:**

A pregnant woman was not diagnosed in a clinic despite being at the verge of giving birth. She was turned away, was on her way to another hospital, and gave birth in a toilet in a dissociative state. The baby survived, but the mother was declared guilty of attempted manslaughter and received a 6-year prison sentence. The expertise of a perinatal psychiatrist reversed the verdict and the court apologized to the mother, now living with her son.

**Conclusions:**

This case shows the severe consequences when pregnancy and labor are not recognized by health professionals. The reversal of the original sentence is considered a pioneer case of restorative justice in the context of unperceived pregnancy and obstetric violence. Health providers and courts need to be informed by perinatal mental health professionals about the impact of unperceived pregnancy and obstetric violence.

## Introduction

Pregnancy denial is a psychopathological condition which is believed to happen in approximately one in every 475 pregnancies (Wessel and Buscher 2002). New research even talks about one in 300 pregnancies (Simermann et al. 2018). During pregnancy denial, which will be referred to as unperceived pregnancy as recommended by the Reproductive Mental Health Forensic Special Interest Group of the International Marcé Society, women are unaware they are pregnant and pregnancy-related symptoms are either absent or attributed to non-pregnancy–related causes. It can occur as a dissociative episode or, very rarely, in the context of an acute and delusional mental disorder. The absence of physical symptoms of pregnancy to such a degree that even those closest to the mothers also fail to perceive changes in her body like weight gain is striking and difficult to comprehend. In one large population-based study, the pregnancy was even missed by doctors in 38% (Wessel and Buscher 2002). According to some authors, the denial works like a psychological mechanism of defense or protection against the anticipated negative consequences of a pregnancy (Wessel and Buscher 2002).

In an unperceived pregnancy, the onset of labor is often mistaken for severe menstrual cramping or food poisoning. Under the stress and trauma of unexpected and unassisted labor, dissociation occurs facilitated by the altered state of consciousness typical of physiological labor (Olza et al. 2020). Labor is usually unassisted, making it risky for the lives of both the newborn and their mothers (Schultz and Bushati 2015). Because the pregnancy is unknown (unperceived), the urge to push is often perceived as a need to defecate; therefore, it is very common that women give birth on the toilet (Meyer and Oberman 2001, Neifert and Bourgeois 2000, Spinelli 2001). In all the cases that have been studied, the story told by the women is similar: not recognizing they are in labor, and always in a state of dissociation so that their ability to perceive the events as they are happening is severely compromised (Barnes & Buist 2020). Unperceived pregnancy is a dissociative disorder with potentially lethal consequences: frequent obstetric complications such as prematurity, severe maternal hemorrhage with a risk of death, and, unfortunately, neonaticide (Simermann et al. 2018).

The latter can happen in the context of an acute stress reaction with dissociative symptoms after having labored alone, unexpectedly, experiencing terror and panic. In an investigation of about 74 neonaticides, 18 had taken place in the toilet: the majority of them in the context of an unperceived pregnancy (Jenkins et al. 2011). The most frequent cause of neonaticide is usually maternal negligence, abandoning the baby in the rubbish or a container without being able to remember the circumstances of the birth. Women can feel tremendously confused and disorientated during labor or may panic after the birth (Jenkins et al. 2011). In some cases, they do not even understand in their dissociative state that they have given birth; in other cases, they believe that the neonate is deceased at birth because of the sensory distortions imposed by their dissociative state. For such reasons, it is essential to identify the risks that leave women more vulnerable to an unperceived pregnancy. It is also critical to raise awareness not only with all health professionals but also with the wider community to prevent these cases in addition to providing competent care for the women who suffer because of an unperceived pregnancy (Klier et al. 2013).

## Material and methods

This brief report describes a mother who did not perceive her pregnancy and was misdiagnosed when she was about to give birth. The subsequent events of an unassisted birth and disposal of a newborn (who survived) led to a sentence of 6 years for the women. The expertise of a perinatal psychiatrist was instrumental in reversing the verdict and the court apologized to the mother who is now living with her son.

This reversal of the original sentence is considered a pioneer case of restorative justice in the context of unperceived pregnancy and obstetric violence.

## Results

This is a report of a 30-year-old woman in Costa Rica with no personal nor familial history of previous mental disorders. She was divorced and on treatment with oral contraceptives, working full time as a cooking assistant and had primary education. Her only health issue was being overweight (BMI > 30). On a day of May in 2016, she went to the hospital due to severe abdominal pain. She was dismissed from the hospital after being prescribed an assortment of medication for a supposed “nonspecific abdominal infection with the presence of an intra-abdominal mass.” Feeling desperate, she asked a friend to take her to another hospital. During the journey, she managed to stop at a bank office where she went into the toilet; she gave birth prematurely to a male neonate, later determined to be born at 6 months of gestation and left him in the toilet’s rubbish bin. The newborn baby was saved thanks to the swift care of the bank workers who alerted the emergency services while talking to the baby to calm him and arranging his transfer and admission into hospital.

The mother was later arrested because of what had been recorded regarding her entrance and exit from the bank. Four months after delivery, she was allowed who meet her son, who had been taken care of by maternal grandparents, and later on she moved in with them and was living a stable life with her son. The child is 5 years old, healthy, in school, and with a secure attachment to his mother according to the social services. At that moment perinatal forensic evaluation was performed online from Spain including psychiatric history, psychiatric examination, and administration of DES (Dissociative Experiences Scale) and ACE (Adverse Childhood Experiences), in both she scored below cut point. A diagnosis of Dissociative Disorder 300.15, DSM-5 or F 44.89, ICD 10 within the context of an unperceived pregnancy was diagnosed. However on February 2021 she was declared guilty of attempted manslaughter with a 6-year prison sentence. An appeal was submitted by the lawyer and public defender for the mother. A forensic perinatal psychiatrist was invited to declare at the Costa Rica consulate in Madrid. On 8 September 2021, the Court of Criminal Appeal of San José, Costa Rica, decided to annul the trial in which a mother had been declared guilty accepting the perinatal psychiatrist’s report as had been proposed by researchers (Barnes 2022).

Recently, a new trial took place; again the perinatal psychiatrist was allowed to testify in Madrid, and it was stated:

The Court agreed that the defendant undoubtedly suffered obstetric violence in the hospital where she was treated on May 5th 2016, where she was not listened to, pointing out the medical negligence suffered in the hospital by not diagnosing her labour nor the pregnancy two hours before she gave birth. Both she and her baby were denied the right to dignified assistance, highlighting that she was an uninsured user. Obviously, if she had not been ignored, neither her baby, family nor herself would have suffered physically and mentally the consequences of the facts. The accused was revictimized: singled out by the media, subjected to a long and tedious criminal process of two trials without any kind of support or accompaniment by state or non-governmental organizations that call themselves defenders of gender violence against women. In this case, they have failed to protect the fundamental right to health of people, the accused and her minor son, in vulnerable conditions.

It was proven in this specific case that the accused acted in a state of traumatic shock, in a dissociative state in which the accused found herself functioning after labor in automatic survival mode; in conditions which prevented her from analyzing appropriately the sensory information she was receiving, without being able to explain or remember -to this date- what happened, according to the evidence that was produced in the prosecution and immediacy of the statement made by the accused. This Chamber perceives in the accused an emotional affectation as a result of this fact. She is responsible with her son, who is currently seven years old, but the memory of what happened has her still immersed in a great sadness. The court considered proven with certainty the innocence of the accused in the act attributed to her by the prosecution and, in the mother was acquitted of all offences and responsibility for the crime of attempted manslaughter to the detriment of her son.

## Discussion and conclusions

This case represents the typical symptomatology of an unperceived pregnancy. Neither the patient nor anyone around her had awareness that she was pregnant. She was taking oral contraceptives and kept bleeding regularly. She was not diagnosed in a gynecological appointment in the first trimester of pregnancy, nor was she even diagnosed the very same day of labor, when she was attended to at a hospital 2 h before giving birth. The mother’s behavior is a reflection of symptomatology typical of dissociation, giving birth in a public place where there are cameras filming her entry and exit to the toilet, getting rid of the newborn, and leaving it without a second thought about the consequences. She acts in a robotic and mechanical manner, reflecting the split between body and mind in a depersonalized mental state, a typical feature of dissociation. As she got back into the car, she fell asleep, so they drove her to her home where she slept for the rest of the day, returning to work as usual the day after. Additionally, she presents with retrograde amnesia and, on knowing what has happened, affirms to not understand how this could have happened to her, even after almost 5 years since the critical event. The mother finds it puzzling, incapable of understanding her own proven conduct as well as feeling immense sadness and guilt for the possibility of unknowingly harming her son whom she loves dearly. As soon as she understood that she had become a mother she began acting with maternal love, calling the hospital to find out about her son, asking her parents to inform her of his progress, offering expressed breastmilk to nurture him, and focusing her life, from that point on, in caring for him and ensuring him a healthy development.

During the trial, the perinatal psychiatrist emphasized that the lack of diagnosis and care during her stay in hospital 2 h before the birth contributed to the events; everything would have been different if the pregnancy and ongoing labor had been diagnosed and she had received care while giving birth. That neglected attention put the lives of both mother and baby at risk. This woman was able to create a healthy and stable bond with her son with whom she currently lives. When the judicial decision was made public, the jury verbally apologized to the mother, expressing how the system had failed her twice: first the medical system, second the legal system. The dissemination of this specific knowledge about unperceived pregnancy, and unassisted and traumatic birth to health professions caring for women in childbearing age is of utmost importance when it comes to pregnancy and peripartum-associated events with fatal or near-fatal outcomes. It is important that all health professionals know unperceived pregnancy can happen and inform women that oral contraceptives can sometimes fail. When a woman presents in labor without knowing she is pregnant she should not be judged nor criticized, but rather cared for and supported to minimize trauma for her and the infant. Mass media should be very careful when reporting such causes to promote education and not judgement and misinformation. With the proper support, most women can be adequate mothers after unperceived pregnancy. The court’s decision to reverse the verdict is considered a pioneer case of restorative justice and furthers the understanding of an unperceived pregnancy as a dissociative disorder (Majety and Bejugam 2016).

## Declarations

The subject gave informed consent to publish her story and the CARE checklist was applied.
